# The inflammatory potential of diet in determining cancer risk; A prospective investigation of two dietary pattern scores

**DOI:** 10.1371/journal.pone.0214551

**Published:** 2019-04-12

**Authors:** Stina Bodén, Robin Myte, Maria Wennberg, Sophia Harlid, Ingegerd Johansson, Nitin Shivappa, James R. Hébert, Bethany Van Guelpen, Lena Maria Nilsson

**Affiliations:** 1 Department of Radiation Sciences, Oncology, Umeå University, Umeå, Sweden; 2 Department of Public Health and Clinical Medicine, Sustainable Health/Nutritional Research, Umeå University, Umeå, Sweden; 3 Cancer Prevention and Control Program, University of South Carolina, Columbia, SC, United States of America; 4 Department of Epidemiology and Biostatistics, Arnold School of Public Health, University of South Carolina, Columbia, SC, United States of America; 5 Connecting Health Innovations LLC, Columbia, SC, United States of America; 6 Wallenberg Centre for Molecular Medicine, Umeå University, Umeå, Sweden; 7 Arcum, Arctic Research Center at Umeå University, Umeå, Sweden; Universita degli Studi di Brescia, ITALY

## Abstract

**Purpose:**

Inflammation-related mechanisms may contribute to the link between diet and cancer. We sought to investigate the inflammatory impact of diet on cancer risk using the Dietary inflammatory index (DII) and an adapted Mediterranean diet score (MDS).

**Methods:**

This population-based, prospective cohort study used self-reported dietary data from the Västerbotten Intervention Programme, including 100,881 participants, of whom 35,393 had repeated measures. Associations between dietary patterns and cancer risk were evaluated using Cox proportional hazards regression. We also used restricted cubic splines to test for potential non-linear associations.

**Results:**

A total of 9,250 incident cancer cases were diagnosed during a median follow-up of 15 years. The two dietary patterns were moderately correlated to each other and had similar associations with cancer risk, predominantly lung cancer in men (DII per tertile decrease: Hazard ratio (HR) 0.81 (0.66–0.99), MDS per tertile increase: HR 0.86 (0.72–1.03)), and gastric cancer in men (DII: 0.73 (0.53–0.99), MDS: 0.73 (0.56–0.96)). Associations were, in general, found to be linear. We found no longitudinal association between 10-year change in diet and cancer risk.

**Conclusion:**

We confirm small, but consistent and statistically significant associations between a more anti-inflammatory or healthier diet and reduced risk of cancer, including a lower risk of lung and gastric cancer in men. The dietary indexes produced similar associations with respect to the risk of cancer.

## Introduction

A third of all cancer-related deaths may be linked to diet [1] and inflammation-related mechanisms may be involved [2]. A pro-inflammatory diet estimated by higher Dietary inflammatory index (DII) scores has been associated with both systemic low-grade inflammation and increased risk of cancers including prostate, breast, colorectal, lung, and pancreas [3–6].

Excess body fat is an established risk factor for many types of cancers [1]. An energy-dense diet may induce weight gain, which can lead to a pro-inflammatory state, but also could increase cancer risk through an altered sex hormone profile [7]. Adherence to a Mediterranean diet, represented by a higher Mediterranean diet score (MDS) [8], has been associated with both lower levels of inflammatory biomarkers and lower risk of several types of cancer [9]. The high dietary intake of antioxidants, including polyphenols, associated with higher adherence to the Mediterranean diet, may inhibit multiple cancer-related biological pathways [10]. Thus, defining a dietary pattern to distinguish between inflammation and other mechanisms by which the diet might influence cancer risk, is desirable.

The aim of this study was to investigate the inflammatory impact of diet in determining cancer risk using two widely used indices, the inflammation-specific DII and an adapted MDS. These dietary indices were examined in 9,250 prospective cancer cases in a population-based cohort of 100,881 participants, including 35,393 individuals with repeated measures ≥10 years apart.

## Methods

### Study cohort and study population

**Fig 1:**
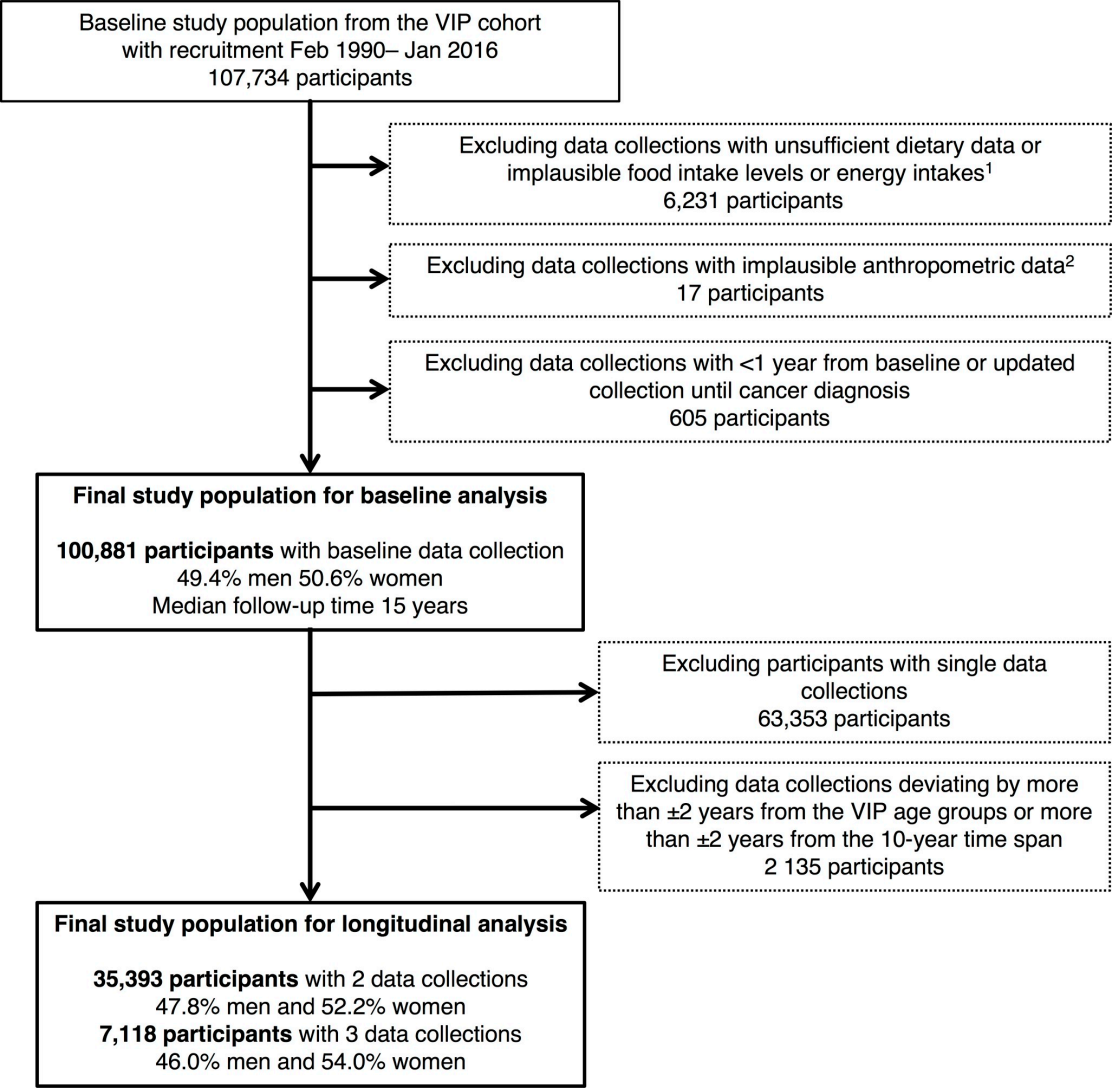
Study design. Illustrating the selection and exclusion of study participants from Västerbotten Intervetion Programme (VIP). ^1^ Implausible food intake levels (FIL): below 1^st^ or above 99^th^ percentile for each version of the food frequency questionnaire (FFQ) and for each sex. Implausible energy intakes: below 1^st^ percentile or >5000 kcal/day ^2^ Implausible anthropometric data: height <130 cm or >210 cm, weight <35 kg or body mass index (BMI) <15 or >70 kg/m^2^.

Study participants were selected from the Västerbotten Intervention Programme (VIP) cohort, an ongoing population-based, prospective cohort in northern Sweden, established in 1986 [11]. During a decennial health examination, residents 40, 50, and 60 years of age (also 30 years during 1990–1996), were asked to complete a questionnaire on diet and lifestyle and to donate a blood sample. This study included 100,881 participants (50.6% women) with data from Feb. 15, 1990 (excluding the first few years of the cohort, with less-standardized FFQs) to Jan. 19, 2016 (Fig 1). All participants were followed until either diagnosis of an invasive cancer or until end of follow-up on Nov. 10, 2016. Exclusion criteria were previous cancer diagnosis other than non-melanoma skin cancer, insufficient dietary data, implausible food intake levels (FIL) (below the 1^st^ or above the 99^th^ percentile for each version of the food frequency questionnaire (FFQ) and for each sex), implausible energy intakes (below the 1^st^ percentile or >5000 kcal/day), implausible anthropometric data (height <130 cm or >210 cm, weight <35 kg or body mass index (BMI) <15 or >70 kg/m^2^), and cancer cases diagnosed within 1 year of their last measurement (n = 605).

We also estimated associations between 10-year changes in dietary pattern scores and cancer risk. Participants with health examinations deviating by more than ±2 years from the VIP age groups or more than ±2 years from the 10-year time span between health examinations, were excluded. For participants with three measurements (n = 7,118), the two earliest measurements were used. After exclusions (n = 2,135), a total of 35,393 participants were included in the longitudinal analyses.

### Dietary data

Dietary data were harmonized and refined by the Northern Sweden Diet Database (NSDD) management. Validated FFQs—a longer version with 84 items and a shortened version of the same FFQ with 64–66 items—were used to calculate dietary pattern scores [12–14]. Food items, reported on a fixed, nine-option scale ranging from never to ≥4 times/day, were converted into daily intakes (g/day) using reported portion sizes combined with data from the National food composition database [15]. For nearly all repeated measures (99.8%), and 67.5% of the baseline measurements, the participants filled out the shorter FFQ.

### Dietary inflammatory index

Detailed descriptions of development and scoring algorithm of the DII [3], as well as construct validations can be found elsewhere [4, 5]. Briefly, nearly 2000 articles investigating the relation between specific dietary factors and six different inflammatory markers (interleukin (IL)-1β, IL-4, IL-6, IL-10, tumor necrosis factor alpha and CRP (C-reactive protein)) were reviewed. A total of 45 specific foods and nutrients were indexed and scored to derive an inflammatory effect score for each parameter. Dietary data were linked to a database including eleven datasets covering most regions of the world, from which means and standard deviations for the 45 food parameters were derived. These parameters were then used as multipliers to express an individual’s exposure relative to the “standard global mean” as a z-score, by subtracting the “standard global mean” from the reported amount and dividing the difference by the standard deviation. The value was converted to a centered proportion score for each food parameter and subject, and multiplied by the corresponding food parameter effect score to produce a food parameter-specific DII score. In this study, 30 of the original 45 foods and dietary components were available for calculation, thus 15 food parameters were lacking (listed in S1 Table), a proportion similar to that observed in other observational studies using the DII [16–18].

### Mediterranean diet score

The Mediterranean diet is characterized by high intake of vegetables, legumes, fruits, nuts, seeds, cereals, and olive oil, moderately high intake of fish, low to moderate intake of dairy products, moderate intake of alcohol, and low intake of saturated fat, meat and meat products [8]. We used an adapted version of the MDS previously applied in Swedish populations based on existing knowledge about positive health effect of whole-grain cereals, moderate alcohol intake, and also that polyunsaturated fatty acids (PUFA) and not only monounsaturated fatty acids (MUFA) are important unsaturated fats in non-Mediterranean countries [19]. The adapted MDS has eight components (listed in S1 Table), 1) vegetables and potatoes, 2) fruit and fresh juices, 3) wholegrain cereals, 4) fish and fish products, 5) ratio of MUFA + PUFA to saturated fat (SFA), 6) alcohol intake, 7) meat and meat products, and 8) dairy products. The intake of each component was adjusted to daily energy intakes of 2500 kcal for men and 2000 kcal for women, using the nutrient density method (e.g., component/total energy). For components 1–6, a value of 1 was assigned to subjects whose consumption was higher than the sex- and FFQ-specific median and 0 for intakes below the median, except for alcohol where participants with intakes <50g/day were assigned 1, and 0 if >50g/day. For meat and dairy products, a value of 1 was assigned for subjects with intakes below the median. The summed MDS ranges from 0 (low adherence) to 8 (high adherence).

### Covariates

Smoking status was classified as daily smoker, ex-smoker (former daily smoker), or never smoker (including occasional smoker and former occasional smoker). Diabetes was defined as self-reported or diagnosed at the health examination according to fasting blood glucose (≥7.0mmol/L) or 2-hour post-load plasma glucose (≥12.2mmol/L in capillary blood). BMI (kg/m^2^) was calculated using measurements taken by a health care professional. Physical activity refers to recreational physical activity, harmonized between questionnaire versions and classified in three levels: low (no recreational physical activity exercise), medium (up to 2 times a week), and high (≥3 times a week). Educational status was defined at three levels; elementary school (including lower secondary, up to 9 years of school), upper secondary school or post-secondary education. Total energy intake was calculated from FFQ-derived dietary data and expressed as kcal/day.

### Identification of cancer cases

Cancer endpoints were identified by linkage to the essentially complete regional branch of the Swedish Cancer Registry. Cases were defined based on ICD-10 codes as first incident malignancy (all types), as well as first incident breast (C50), prostate (C61), lung (C34), gastric (C16), pancreas (C25), colorectal (C18-C20.9), and gastrointestinal (GI) including: esophagus (C15), gastric (C16), liver/intrahepatic bile ducts (C22), pancreas (C25) and small intestine (C17) cancer. We also investigated smoking-related and obesity-related cancers. Smoking-related cancers were defined according to the International Agency for Research on Cancer (IARC) [20]. Tumor sites for which evidence of a link to tobacco smoking is suggested to be sufficient, are: lip/oral cavity/pharynx (C00-C14), liver/intrahepatic bile ducts, larynx/trachea/bronchus/lung (C32-C34), cervix (C53, D06), colorectum, kidney (C64), esophagus, pancreas, stomach, urinary bladder (C67), as well as acute and chronic myeloid leukemia (C91-95 and D46, excluding C91.4)[20]. Obesity-related cancers were defined as cancer of the esophagus, gastric, colorectum, liver, gallbladder (C23-24), pancreas, breast (post-menopausal, approximated as breast cancers diagnosed after the age of 55 years), endometrium (C54), ovary (C56), kidney, meningioma (C70.0), thyroid (C73), and multiple myeloma (C90.0) [7]. Non-smoking-related and non-obesity-related cancers were defined as all other cancers not included in these definitions.

### Ethics

This study was approved by the Regional ethical review board of northern Sweden (Dnr 2013/332–31). All study subjects provided written informed consent at recruitment for all collection for research purposes, and the study was conducted in accordance with the Declaration of Helsinki.

### Statistical analyses

Baseline characteristics of men and women were calculated for sex- and FFQ-specific categories approximating tertiles of the dietary pattern scores. DII tertiles (T) were constructed according to the distribution of participants. MDS tertiles were distributed to avoid ties: T1) Score 0–3, T2) Score 4, and T3) Score 5–8. Comparisons were made using Pearson Chi-square tests for categorical variables and ANOVA for continuous variables. Correlations between dietary patterns were estimated with Spearman’s correlation coefficient.

Associations between baseline dietary patterns and cancer risk were evaluated using Cox proportional hazards regression with age as the time scale. The proportional hazards assumption was checked by evaluating Schoenfeld residuals. In the all-cancer risk analysis, sex showed signs of non-proportionality characterized as a higher risk of cancer in women compared to men before age 67 years and the opposite after age 67. Therefore, risk estimates are presented for all participants, stratified by sex within the Cox model, but also for men and women separately. In the analysis of breast and lung cancer, both BMI and smoking showed signs of non-proportionality. Because stratification for BMI categories or smoking status did not affect risk estimates, estimates from non-stratified models are presented.

To facilitate comparisons between risk estimates, linear associations are presented as hazard ratios (HR) per tertile decrease in DII or tertile increase in MDS, obtained by modelling continuous scaled variables, i.e. by dividing each dietary pattern score by its respective sex- and FFQ-specific intertertile range. The mean intertertile ranges were 1.7 for DII and 2 for MDS. Estimates were adjusted for covariates with a potential association to both dietary pattern and cancer risk: energy intake, BMI, physical activity, smoking, and educational status. In sensitivity analyses, HRs were estimated separately by age groups (30–40, 50, and 60 years), smoking status (non smokers, ever smokers), and BMI (BMI >30kg/m^2^, BMI <30kg/m^2^). HRs were also estimated by excluding participants with diabetes. Heterogeneity in HR estimates between subgroups were tested with a Wald’s test.

To test for non-linear associations, continuous dietary pattern variables were modelled using restricted cubic splines (with knots at the 5^th^, 50^th^, and 95^th^ percentiles). Tests for associations were made with a likelihood ratio test comparing the dietary pattern spline model with a model without the dietary pattern. Non-linearity was tested with a likelihood ratio test comparing the spline model to a linear model.

To assess the predictive accuracy of the dietary patterns, we estimated Harell’s C-index in Cox-models using the baseline measurement. C-indices were calculated using ten-fold cross-validation to avoid overfitting.

We evaluated longitudinal associations between dietary patterns and cancer risk by fitting Cox models with start of follow-up 1 year after the repeat measurement, using age as the time scale. Participants were classified as “Unchanged healthy”, “Changed unhealthy to healthy”, “Changed healthy to unhealthy”, or “Unchanged unhealthy” according to baseline and repeat values on dichotomous dietary pattern variables (“unhealthy” defined as DII T3 and MDS T1, using sex- and FFQ-specific cut-offs). We also evaluated longitudinal associations between continuous change in dietary pattern score (Δ = repeat–baseline) and cancer risk. HRs per tertile decrease in ΔDII or tertile increase in ΔMDS were obtained by modelling continuous scaled difference variables (i.e., by dividing each Δ-variable by their respective sex- and FFQ specific intertertile ranges) in Cox models. Estimates were adjusted for baseline and Δenergy intake, baseline and ΔBMI, smoking (non-smoker, ex-smoker, stopped smoking, started smoking, continued smoking), physical activity (unchanged, decreased less activity, more physical activity), and baseline educational status.

All computations were conducted in R v.3.4.2 (R Foundation for Statistical Computing, Vienna, Austria). All tests were 2-sided, and P-values <0.05 were considered statistically significant.

## Results

### Baseline characteristics

Characteristics of the 100,881 study participants at first visit are presented in Table 1. Mean age at baseline increased across tertiles of MDS from 45.2 to 48.0 years for men (*P* <0.001) and from 44.3 to 48.7 for women (*P* <0.001). Mean age was more similar across DII tertiles, though *P* <0.001. Obesity was more common among men with a more pro-inflammatory diet as estimated by the DII (*P* <0.001), and among both men and women with low adherence to MDS (*P* <0.001). Participants with higher DII and lower MDS scores (i.e. more pro-inflammatory/unhealthier diet), were less likely to be married or co-habitating, to have post-secondary education or to be physically active, but more likely to be current smokers (*P* <0.001 for all). Additionally, they were less likely to have been diagnosed with diabetes (DII men *P* = 0.007, DII women *P =* 0.03, MDS men *P*<0.001, MDS women *P*<0.001).

**Table 1:** Baseline characteristics by tertiles of DII and MDS for men (n = 49,880) and women (n = 51,001) in the VIP.

	DII				MDS				
Men	T3^a^	T2	T1	*P*^b^	T1^a^	T2	T3	*P*^b^	Missing
Proportion of participants, n (%)	16626 (33.3)	16628 (33.3)	16626 (33.3)		19830 (39.8)	11666 (23.4)	18384 (36.9)		
Dietary score, min,max	5.53,1.47	2.12, -0.30	0.33, -5.25	<0.001	0,3	4	5,8	<0.001	-
Age, mean±sd	46.5±9.00	46.9±9.05	46.5±9.09	<0.001	45.2±9.29	46.9±9.06	48.0±8.52	<0.001	-
Obese (BMI ≥30), n (%)	2616 (15.7)	2451 (14.7)	2290 (13.8)	<0.001	3048 (15.4)	1757 (15.1)	2552(13.9)	<0.001	-
Not married/co-habitating^c^, n (%)	4090 (24.6)	3209 (19.3)	2881 (17.3)	<0.001	4554 (23.0)	2414 (20.7)	3212 (17.5)	<0.001	377 (0.8)
No post-secondary education, n (%)	13517 (80.7)	12258 (73.7)	11496 (69.1)	<0.001	15848 (79.9)	8695 (74.5)	12638 (68.7)	<0.001	281 (0.6)
Diabetes, n (%)	744 (4.5)	750 (4.5)	867 (5.2)	0.007	777 (3.9)	560 (4.8)	1024 (5.6)	<0.001	157 (0.3)
Current smoker, n (%)	3120 (18.8)	2397 (14.4)	1933 (11.6)	<0.001	3337 (16.8)	1787 (15.3)	2326 (12.7)	<0.001	720 (1.4)
Low physical activity, n (%)	7857 (47.3)	6638 (39.9)	5395 (32.4)	<0.001	9013 (45.5)	4644 (39.8)	6233 (33.9)	<0.001	756 (1.5)
**Women**									
Proportion of participants, n (%)	16999 (33.3)	17001 (33.3)	17001 (33.3)		21148 (41.5)	11472 (22.5)	18381 (36.0)		
Dietary score, min,max	5.35,1.82	2.14,0.14	0.50, -5.06	<0.001	0,3	4	5,8	<0.001	-
Age, mean±sd	46.3±9.10	46.6±9.00	46.3±9.00	<0.001	44.3±8.96	46.6±8.85	48.7±8.59	<0.001	-
Obese (BMI ≥30), n (%)	2519 (14.8)	2476 (14.6)	2576 (15.2)	0.310	3279 (15.5)	1706 (14.9)	2586 (14.1)	<0.001	-
Not married/co-habitating^c^, n (%)	3693 (21.7)	2914 (17.1)	2709 (15.9)	<0.001	4039 (19.1)	2152 (18.8)	3125 (17.0)	<0.001	418 (0.8)
No post-secondary education, n (%)	12209 (71.8)	10967 (64.5)	10135 (59.6)	<0.001	14608 (69.1)	7397 (64.5)	11506 (62.6)	<0.001	361 (0.7)
Diabetes, n (%)	544 (3.2)	521 (3.1)	585 (3.4)	0.032	613 (2.9)	375 (3.3)	662 (3.6)	<0.001	210 (0.4)
Current smoker, n (%)	3951 (23.2)	2830 (16.6)	2169 (12.8)	<0.001	4154 (19.6)	1996 (17.4)	2800 (15.2)	<0.001	426 (0.8)
Low physical activity, n (%)	7879 (46.3)	6449 (37.9)	5242 (30.8)	<0.001	9291 (43.9)	4240 (37.0)	6039 (32.9)	<0.001	723 (1.4)

^a^ Represents a more pro-inflammatory diet (DII) or poor adherence to MDS.

^b^ P values were determined using ANOVA for continuous variables and Chi Square test for categorical variables.

^c^ Based on the civil status questionnaire alternatives single, separated, widow or widower.

Abbreviations: DII, dietary inflammatory index; MDS, Mediterranean diet score; T, tertile; VIP, Västerbotten Intervention Programme.

Baseline DII and MDS scores were moderately negatively correlated (r = -0.34, *P*<0.001) and correlations were similar for the repeat measures (S2 Table).

### Baseline associations between dietary patterns and cancer risk

**Fig 2:**
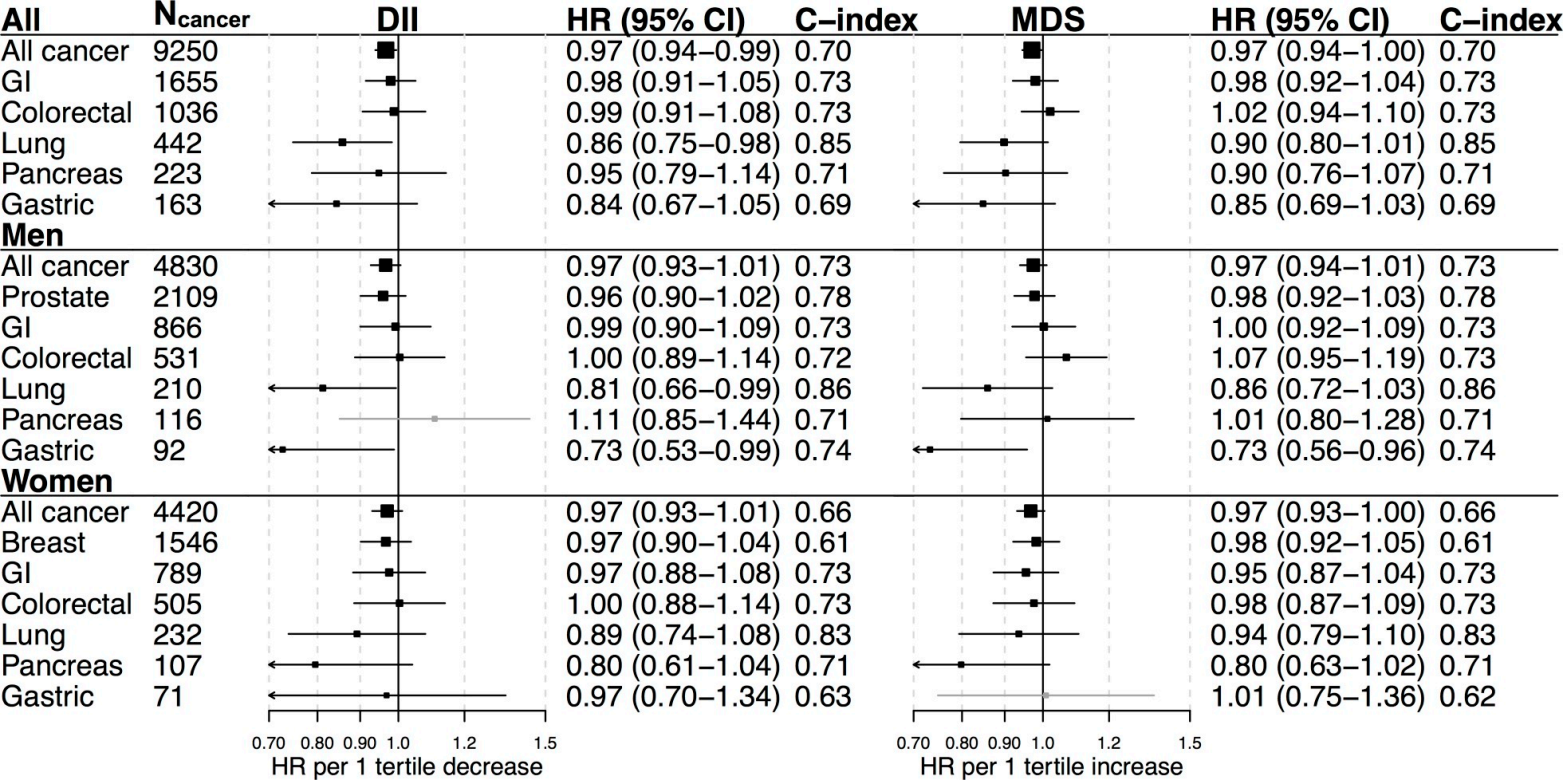
Hazard Ratios (HRs) and 95% CI for cancer per tertile decrease in DII, and tertile increase in MDS, at baseline in all participants (n = 100,881), men (n = 49,880), and women (n = 51,001) in the VIP. HRs obtained from Cox regression using age as the time scale. Dietary pattern variables were included as continuous variables scaled by dividing by the sex and FFQ-specific intertertile ranges. Mean intertertile range were: DII = 1.7, MDS = 2. Estimates marked in gray had a potential non-linear association, illustrated in Fig 3. Estimates were adjusted for energy intake, BMI, physical activity, smoking, educational status, and, in the all participant analyses, stratified by sex in the Cox model. Predictive accuracy (C-index) for cancer for each model were calculated using predictions with the Cox regression models using ten-fold cross-validation. The C-index is measured on a scale from 0.5 to 1, where 0.5 corresponds to a prediction accuracy no better than guessing and 1 corresponds to perfect prediction.

During follow-up (median 15 years), 9,250 prospective cancer diagnoses were detected, 4,830 in men and 4,420 in women. Linear HRs for cancer by baseline dietary pattern scores, adjusted for potential confounders, are presented in Fig 2. Lower DII, and higher MDS, were weakly associated with a lower risk of cancer (HR (95% CI) per tertile decrease in DII: 0.97 (0.94–0.99), HR per tertile increase in MDS: 0.97 (0.94–1.00)). DII was associated with reduced risk of lung cancer, which was statistically significant in men (HR per tertile decrease in DII in men: 0.81 (0.66–0.99), in women: 0.89 (0.74–1.08)). Both DII and MDS scores were associated with reduced risk of gastric cancer in men (HR per tertile decrease in DII: 0.73 (0.53–0.99), HR per tertile increase in MDS: 0.73 (0.56–0.96). Neither dietary pattern was associated with risk of prostate cancer in men, breast cancer in women, or GI, colorectal and pancreas cancer in both sexes (Fig 2).

The overall accuracy for predicting cancer for models including age, energy intake, BMI, physical activity, smoking, educational status, and dietary patterns, was similar for the two dietary patterns (C-index = 0.70, Fig 2) and slightly better in men compared to women (C-index 0.73 and 0.66, respectively). None of the dietary patterns markedly improved the prediction accuracy for overall or site-specific cancer risk in either sex. C-index was unmodified when excluding energy intake, for a model limited to variables easily obtainable in a clinical or internet-based “risk calculator” type of setting. Excluding participants with diabetes in sensitivity analyses in this study did not affect the results (S1 Fig).

Associations between dietary patterns and overall cancer risk in subgroups defined by baseline age, smoking status, and BMI, are presented in S2 Fig. HRs were generally similar across subgroups. In men, the association between DII score and cancer risk appeared stronger in participants aged 30 and 40 years (HRs per tertile decrease in DII: 0.89 (0.80–0.99), but the test of heterogeneity was not statistically significant (*P*_heterogeneity_ = 0.28).

For smoking-related cancers, lower DII or higher MDS were mainly associated with a decreased risk in ever smokers, with weak evidence of heterogeneity in associations between smoking-related and other cancers (*P*_heterogeneity_ = 0.12 and 0.03 for ever smokers and non-smokers, respectively (S3 Fig). In contrast, for non-smoking-related cancers, i.e. all cancer sites not included in the group of smoking-related sites, lower DII was associated with a decreased risk in non-smokers, and not in ever smokers (*P*_heterogeneity_ = 0.13). There were no clear differences in the relation between the risk of obesity- or non-obesity-related cancer and dietary patterns.

**Fig 3:**
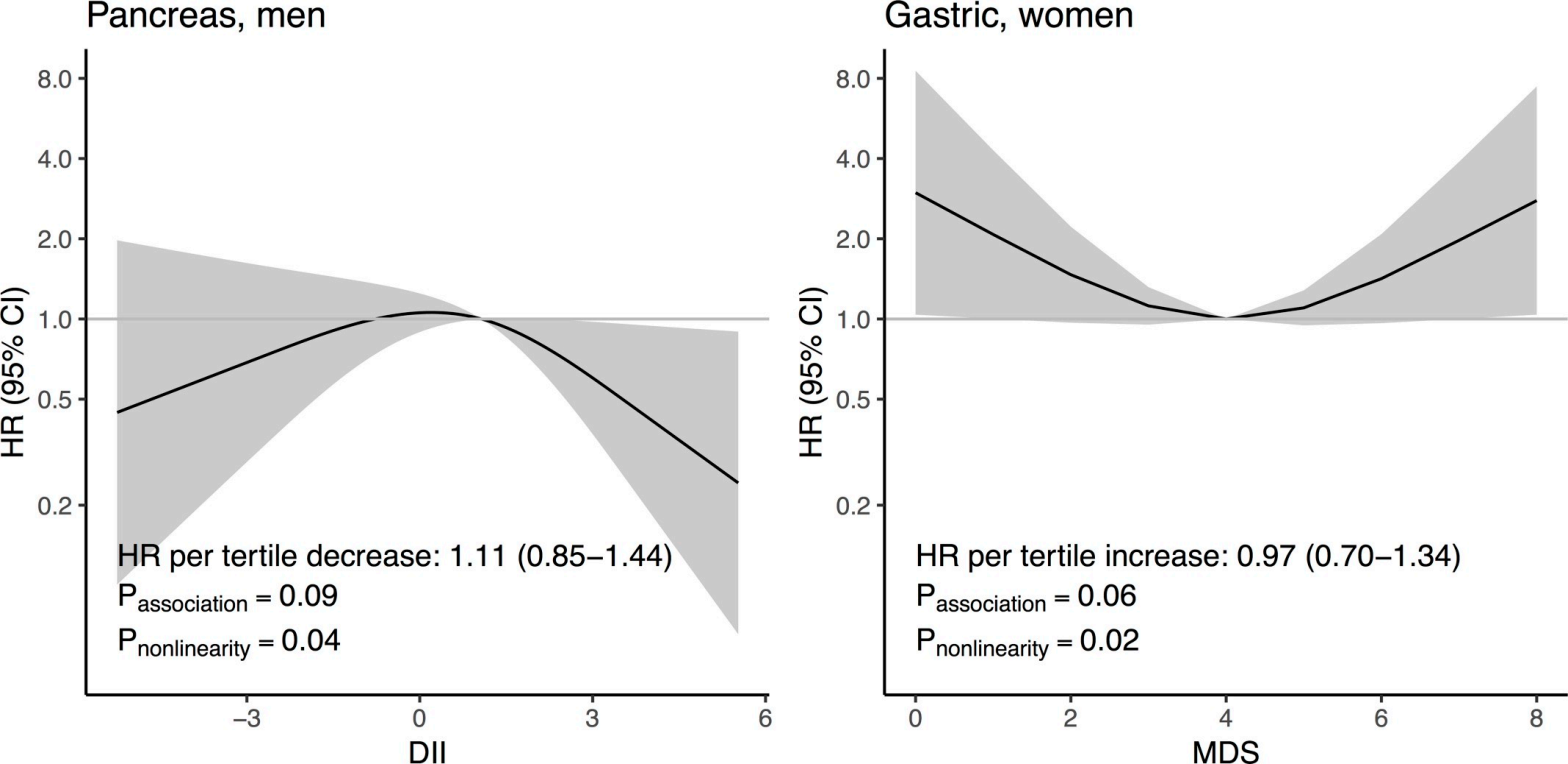
Hazard Ratios (HRs) (black line) and 95% confidence interval (CI) (gray area) of pancreas cancer in men by DII, and gastric cancer in women by MDS. HRs were calculated with restricted cubic splines (with knots on the 5^th^, 50^th^, and 95^th^ percentiles) in Cox regression models using attained age as time scale. Presence of an association were tested with a likelihood ratio test comparing the dietary pattern spline model with a model without dietary pattern. Nonlinearity was tested with a likelihood ratio test comparing the spline model to a linear model. The HRs were adjusted for energy intake, BMI, physical activity, smoking, and educational status.

HRs for cancer types by DII and MDS in all participants, men, and women, modelled by restricted cubic splines, are presented in S4 Fig. Linear associations could be assumed for all associations except DII and pancreatic cancer risk in men, and MDS and gastric cancer in women (*P*_nonlinearity_ = 0.04 and 0.02, respectively), presented separately in Fig 3. The association for pancreas cancer manifested as a possible lower risk in men with high, and to a lesser extent low, DII compared to the median (*P*_association_ = 0.09). The suggested nonlinear association between MDS and gastric cancer risk in women was U-shaped, with increased risk at low or high MDS compared to the median (*P*_association_ = 0.06).

### Longitudinal associations between dietary patterns and cancer risk

Moderate correlations were observed between the baseline and repeat measurement for each dietary pattern (r = 0.40 to 0.53) (S2 Table). Most participants remained in the same tertile of dietary pattern distribution over the 10-year period (S5 Fig).

**Fig 4:**
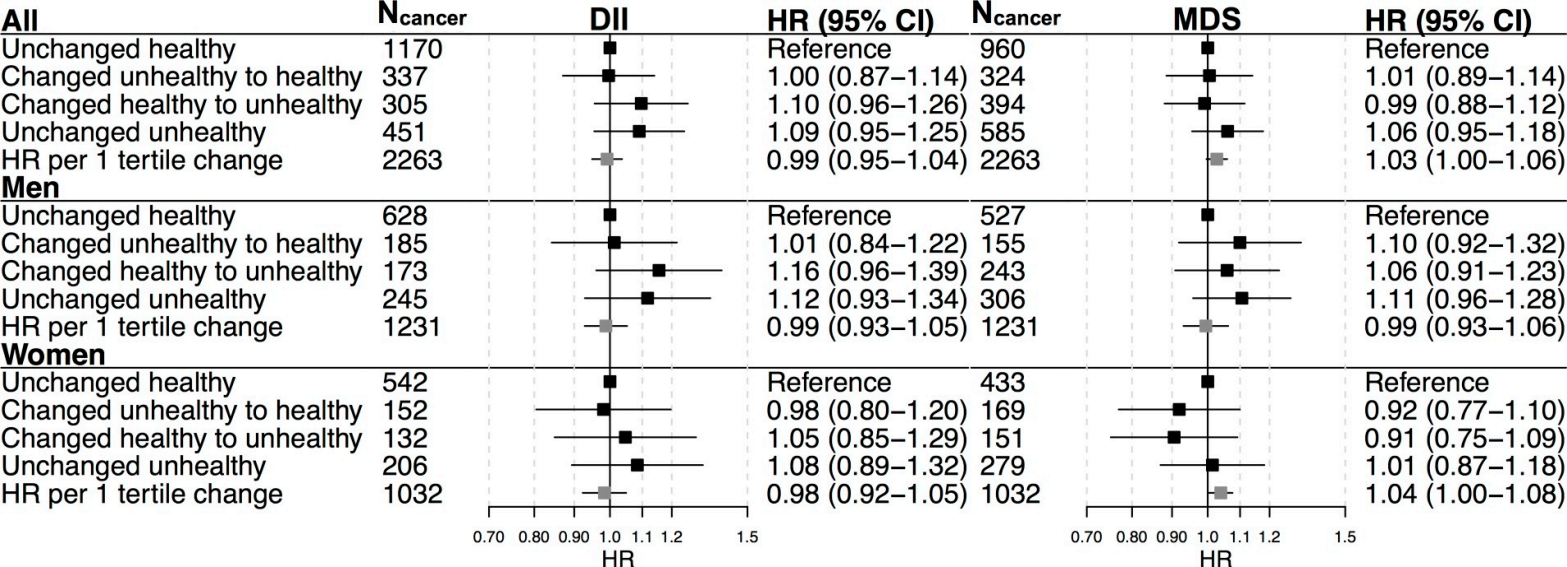
Hazard Ratios (HRs) and 95% confidence interval (CI) for cancer per tertile or category of 10-year change (Δ) in dietary patterns DII and MDS estimated in VIP participants with repeat measurements (n = 35,393). HRs were obtained from Cox regression using age as time scale, with start of follow-up 1 year after the repeat measurement. Categorical variables were defined according to baseline and repeat values on dichotomous dietary pattern variables (“unhealthy” defined as DII 3^rd^ tertile, MDS 1^st^ tertile, using sex- and FFQ specific cut-offs). HRs per tertile change (decrease in ΔDII, and increase per ΔMDS) were calculated by modelling continuous Δ-variables scaled by dividing by the intertertile range (mean intertertile ranges: ΔDII = 1.3, ΔMDS = 1.5). Estimates were adjusted for baseline and Δenergy intake, baseline and ΔBMI, smoking (baseline non-smoker, baseline ex-smoker, stopped smoking, started smoking, or continued smoking), physical activity (unchanged, less activity, more physical activity), and baseline educational status.

Participants, primarily men, with an unchanged, more pro-inflammatory diet at follow-up, as well as participants who went from “healthy” to a more pro-inflammatory diet over the 10-year period were at a slightly increased risk for cancer; however, the association was attenuated and not significant after adjusting for change in BMI and smoking status (Fig 4). A similar pattern was observed for MSD in men, but the association also attenuated and was not significant in the multivariable model.

Ten-year change in DII was not associated with the risk of cancer (HR per tertile decrease in ΔDII: 0.99 (0.95–1.04) (Fig 4). Participants with greater ΔMDS had a slight increased risk of cancer (HR per tertile increase in ΔMDS: 1.03 (1.00–1.06)). Although the sample size was insufficient to detect heterogeneity between cancer types, the finding appeared to be driven primarily by breast cancer in women (S3 Table).

## Discussion

In this prospective, population-based study, the DII and the MDS were moderately correlated to each other and produced similar associations with the risk of cancer. An anti-inflammatory or healthier diet was weakly associated with a reduced overall cancer risk, most evident for lung and gastric cancer. Ten-year change in dietary pattern score was not related to cancer risk.

These results are consistent with previously observed positive associations between the inflammatory potential of diet and risk of gastric cancer [21, 22], and the inverse association with a Mediterranean diet [23]. Given the divergent incidence trends for specific subtypes of cancer in the upper gastrointestinal tract [22, 24], further investigation including data on anatomical location of the tumor, histological subtype and *Helicobacter pylori* infection in relation to diet are warranted [25]. Our null results for colorectal cancer were surprising, given the wealth of evidence for a role of diet quality in determining risk [23, 26, 27]. Potential associations between diet and any cancer are likely to be mediated in part by body fatness [7]. However, in the present study, “obesity-related cancer”, demonstrated no clear association with dietary indices. Removing BMI from the model did not change the risk estimates and thus, mediation by body fatness is unlikely to entirely explain the null results. Both consumption of foods generally considered unhealthy and total energy intake are underreported to a greater degree by obese compared to non-obese people [28], which might bias potential associations between diet and obesity-related cancers toward the null.

We observed a general association between a more anti-inflammatory/healthier diet and lower risk of lung cancer, consistent with previous findings [6, 23, 29]. Effect sizes were similar in men and women, but the association for DII score was statistically significant in men only. A plausible explanation for associations found between the dietary patterns and “smoking-related cancer” in ever smokers might be a synergistic effect of smoking and unhealthy dietary habits that increases low-grade chronic inflammation, as previously shown for lung cancer [30, 31].

The null findings for prostate and breast cancer contrast with results from meta-analyses of DII [6, 23]. However, there is considerable inconsistency in results for dietary patterns in relation to these cancer types in previous studies conducted in Nordic populations [32–34]. For breast cancer, the strong risk conferred by reproductive factors, which we were unable to adjust for, might explain the fairly weak and inconsistent results for the DII [35, 36].

The study population did not alter its dietary habits substantially according to our supplementary analysis of longitudinal changes in DII and MDS, which probably explains the fairly consistent results for the longitudinal analyses compared to baseline. Interestingly, a change toward better adherence to the MDS, was associated with an increased cancer risk, primarily in women. This might be due to residual confounding by socioeconomic status, not sufficiently captured by the education variable. Higher socioeconomic status is a risk factor for breast cancer, probably acting as a summary marker for factors related to reproduction [37]. Diabetes also was disproportionately common among those with healthier diet and reverse causality due to disease-related dietary changes cannot be excluded [38]. However, excluding participants with diabetes in sensitivity analyses in this study had no material effect on the results.

The fifteen DII food parameters lacking in this study are all considered anti-inflammatory, which might limit the ability of the score to capture an anti-inflammatory diet. However, the range of DII scores in our population is similar to a validation study in an American population based on 44 of the 45 components, which showed a direct association with CRP levels [4].

Nutrients and food components with evidence for a relation to cancer risk are largely covered by both DII and MDS, which undoubtedly contributed to the similar estimates for cancer risk. Whereas the DII was designed specifically to estimate the inflammatory potential of diet [3], the MDS may also capture other mechanisms involved in carcinogenesis, such as reduced free radical production [39] and metabolic function [40]. For example, sugary foods, which can influence blood glucose control and body fatness [40], are considered directly in the MDS but are included only in the broader category of carbohydrates in the DII. Also, red and processed meats, included in the MDS meat component, but not DII, with its high content of salt, N-nitroso, heterocyclic amines, and heme iron have all been implicated in carcinogenesis [41]. Although inflammation may be a common factor in our findings and a major player in explaining the link between diet and cancer, other mechanisms also may be involved.

A weakness in this study is the self-reported dietary intake, which is subject to recall bias and underreporting. Underreporting of socially undesirable foods has been documented, especially in women [42] and obese people [28], and constitutes a possible bias. However, the FFQs had acceptable reproducibility and a validity similar to FFQ measurements in other prospective cohort studies [12–14]. The DII is constructed on a continuous scale, whereas the MDS comprises a number of food groups. Thus, approximate tertiles were used to balance between statistical power and dispersion for the specific cancer-sites. The MDS used in this study was adapted for the northern Swedish population in this study [19], and it is thus not fully representative of the traditional Mediterranean diet. For example, since PUFA make up a substantially larger portion of the unsaturated fatty acid intake in the Nordic diet than in the traditional Mediterranean diet [8], the sum of MUFA and PUFA, rather than MUFA alone, was used in the ratio to SFA. Adaptations of the MDS have been successfully applied in various non-Mediterranean populations [43].

Although confounders may differ between cancer types, we applied the same set of covariates in all analyses, in order to simplify interpretation of results. Information about some potential confounders was lacking, such as use of nonsteroidal anti-inflammatory drugs (NSAID), of particular relevance for CRC, and menopausal hormone therapy, of relevance for breast cancer. Many types of cancer demonstrate substantial intertumoral heterogeneity. More specific anatomic location for cancers of the upper and lower gastrointestinal tract, as well as tumor characteristics, such as histological subtype for lung and gastric cancer, hormone receptor status for breast cancer, and microsatellite instability and other molecular traits in CRC could, therefore, add valuable information.

A major strength of this study is its prospective design, with over 100,000 participants and up to 26 years of follow-up. Because exposure data were collected before cancer diagnosis, reverse causality and disease-specific recall bias were unlikely to have influenced the results. Furthermore, repeated measures (10-year intervals) were available for over 35,000 participants, allowing investigation of longitudinal dietary changes in relation to cancer risk. Although these analyses were sufficiently powered to examine overall cancer risk, a larger sample size would be necessary for site-specific cancer. Additionally, with restricted cubic spline models we could show that most associations were linear. Another important strength is the population-based nature of the cohort used, as demonstrated by the very similar cancer incidence in the VIP and the background population [44], as well as the high participation rate (52–73% over the recruitment period) and the low potential for selection bias [45].

In conclusion, in this prospective cohort study, we confirm small, consistent, and statistically significant associations between a more anti-inflammatory or healthier diet and reduced risk of cancer, for lung and gastric cancer in specific, and particularly in men. Although several mechanisms may be involved, the consistency of the findings for the DII, designed specifically to capture the inflammatory impact of diet, and the MDS, suggests that inflammation may be a common denominator.

## Supporting information

S1 Table Food parameters in adapted DII and adapted MDS. (DOCX)

S2 Table Spearman’s correlations between DII and MDS at baseline, repeat, and across baseline and repeat measurements. (DOCX)

S3 Table Hazard ratios (HRs) and 95% confidence interval (CI) for longitudinal change in DII and MDS for all cancer. (DOCX)

S1 Fig Sensitivity analysis excluding participant diagnosed with diabetes. Hazard ratios (HRs) and 95% CI for all cancer per tertile decrease in DII and tertile increase per MDS at baseline.
(DOCX)

S2 Fig Hazard ratios (HRs) and 95% confidence interval (CI) for all cancer per tertile decrease in DII and tertile increase per MDS at baseline in subgroups defined by age of study entry (VIP age groups ±2), smoking status, and BMI. (DOCX)

S3 Fig Hazard ratios (HRs) and 95% confidence interval (CI) for smoking-related and obesity-related cancer per tertile decrease in DII and tertile increase per MDS at baseline in subgroups defined by smoking status and BMI. (DOCX)

S4 Fig **Restricted cubic splines with hazard ratio (HR) and 95% confidence interval of cancer in a) all participants b) men, and c) women by baseline dietary pattern score**.
(DOCX)

S5 Fig Distribution of 10-year longitudinal changes in dietary patterns. (DOCX)

## Abbreviations

CRC: colorectal cancer
CRP: C-reactive protein
DII: dietary inflammatory index
FFQ: food frequency questionnaire
FIL: food intake level
GI: gastrointestinal
IL: interleukin
MDS: Mediterranean diet score
NSAID: nonsteroidal anti-inflammatory drugs
NSDD: Northern Sweden Diet Database
T: tertile
VIP: Västerbotten Intervention Programme
