# Supplementary material for: The inflammatory potential of diet in determining cancer risk; A prospective investigation of two dietary pattern scores
Source: PLoS One. 2019 Apr 12;14(4):e0214551. doi: 10.1371/journal.pone.0214551 (PMC6461253; doi:10.1371/journal.pone.0214551)
Supplement: S1 Table — (DOCX) [file pone.0214551.s001.docx]

**S1 Table.** Food parameters in adapted DII and food components in adapted MDS.

| **Adapted Dietary inflammatory index (DII)** | | **Adapted Mediterranean diet score (MDS)** | |
| --- | --- | --- | --- |
| Food parameters included in the DII (n=30) calculated from validated FFQ in VIP ranked from most pro-inflammatory to most anti-inflammatory according to the index^a,b^ | | Food groups included in the adapted MDS (n=8) indicating degree of adherence to a Mediterranean diet, calculated from validated FFQ in the VIP^a,c^. The original MDS included 9 food groups^d^ | |
| **Pro-inflammatory (n=9)**  (Raw inflammatory effect score >0)^e^ | **Anti-inflammatory (n=21)**  (Raw inflammatory effect score<0)^e^ | **Unfavorable food groups (n=2)**  If intake above the median in the study population a value of 0 is assigned and 1 if below the median. | **Favorable food groups (n=6)**  If intake above the median in the study population a value of 1 is assigned if above the median and 0 if below the median, except for alcohol where an intake <50g/day is assigned a value of 1. |
| (+) Saturated fat | (-) MUFA | (+) Dairy products | (-) Vegetables and potatoes |
| (+) Total fat | (-) Vitamin B2 | (+) Meat and meat products | (-) Fruit and juices |
| (+) Trans fatty acids | (-) Caffein |  | (-) Fish and fish products |
| (+) Energy | (-) n-6 fatty acids |  | (-) MUFA+PUFA/SFA-ratio |
| (+) Dietary cholesterol | (-) Folic acids |  | (-) Whole-grain cereals |
| (+) Vitamin B12 | (-) Selenium |  | (-) Alcohol (<50g/day) |
| (+) Carbohydrates | (-) Niacin |  |  |
| (+) Iron | (-) Alcohol |  |  |
| (+) Protein | (-) Zink |  |  |
|  | (-) PUFA |  |  |
|  | (-) Vitamin B6 |  |  |
|  | (-) Vitamin A |  |  |
|  | (-) Vitamin C |  |  |
|  | |  | |
| ^a^ Abbreviations: FFQ, food frequency questionnaire; VIP, Västerbotten Intervention Programme  ^b^ Original DII food parameters not included in this study:  Rosemary, Thyme/Oregano, Pepper, Anthocyanidis, Saffron, Eugenol, Flavonones, Onion, Garlic, Flanan-3-ol, Ginger, Flavonols, Isoflavones, Flavones, Turmeric (n=15)  ^c^ Adapted version of MDS based on existing knowledge about positive health effect of whole-grain cereals (*Flight et al 2006*), moderate alcohol intake (*Mukamal et al 2010*) and also that PUFA and not only MUFA are the principal unsaturated fats in a non-Mediterranean diet (*de Lorgeril et al 1994*).  ^d^ MDS on a 9-point scale (0-9) according to *Trichopoulou et al 2003*: Favorable foods (assigned a value of 1 if above the median): vegetables, legumes, fruits & nuts, cereals, fish (n=5). Unfavorable foods (assigned a value of 1 if below the median): dairy products, meat, poultry (n=3)  Alcohol favorable, assigned a value of 1 if consumed between 10 and 50 g per day and to women who consumed between 5 and 25 g per day (n=1)  ^e^ According to the construction of DII originally described by *Shivappa et al 2014*. | | | |
