# Supplementary material for: The inflammatory potential of diet in determining cancer risk; A prospective investigation of two dietary pattern scores
Source: PLoS One. 2019 Apr 12;14(4):e0214551. doi: 10.1371/journal.pone.0214551 (PMC6461253; doi:10.1371/journal.pone.0214551)
Supplement: S2 Table — (DOCX) [file pone.0214551.s002.docx]

**S2 Table.** Spearman’s correlations between DII and MDS at baseline, repeat, and across baseline and repeat measurements*.

|  |  |  | **Baseline** |  | **Repeat** |
| --- | --- | --- | --- | --- | --- |
|  |  | **DII** | **MDS** | **DII** | **MDS** |
|  | **DII** | 1.00 |  |  |  |
| **Baseline** | **MDS** | -0.34 | 1.00 |  |  |
|  | **DII** | 0.53 | -0.21 | 1.00 |  |
| **Repeat** | **MDS** | -0.19 | 0.40 | -0.35 | 1.00 |
| * All correlations significant at level P <0.001 | | | | | |
