# Supplementary material for: The inflammatory potential of diet in determining cancer risk; A prospective investigation of two dietary pattern scores
Source: PLoS One. 2019 Apr 12;14(4):e0214551. doi: 10.1371/journal.pone.0214551 (PMC6461253; doi:10.1371/journal.pone.0214551)
Supplement: S3 Table — (DOCX) [file pone.0214551.s003.docx]

| **Cancer** | **Exposure** | **RR_type** | **N_all** | **RR_all** | **P_all** | **N_men** | **RR_men** | **P_men** | **N_women** | **RR_women** | **P_women** |
| --- | --- | --- | --- | --- | --- | --- | --- | --- | --- | --- | --- |
| All Cancer | DII | per 1 tertile change | 2263 | 0.99 (0.95-1.04) | 0.71 | 1231 | 0.99 (0.93-1.05) | 0.71 | 1032 | 0.98 (0.92-1.05) | 0.62 |
| Prostate | DII | per 1 tertile change | 583 | 0.96 (0.88-1.05) | 0.37 | 583 | 0.96 (0.88-1.05) | 0.37 |  |  |  |
| Breast | DII | per 1 tertile change | 407 | 1.03 (0.93-1.14) | 0.61 |  |  |  | 407 | 1.03 (0.93-1.14) | 0.61 |
| GI | DII | per 1 tertile change | 378 | 0.99 (0.89-1.10) | 0.82 | 215 | 0.98 (0.84-1.14) | 0.76 | 163 | 0.97 (0.83-1.14) | 0.71 |
| CRC | DII | per 1 tertile change | 234 | 1.01 (0.88-1.16) | 0.92 | 130 | 0.99 (0.82-1.21) | 0.95 | 104 | 1.01 (0.82-1.24) | 0.94 |
| All Cancer | MDS | per 1 tertile change | 2263 | 1.03 (1.00-1.06) | 0.09 | 1231 | 0.99 (0.93-1.06) | 0.87 | 1032 | 1.04 (1.00-1.08) | 0.03 |
| Prostate | MDS | per 1 tertile change | 583 | 0.99 (0.90-1.09) | 0.82 | 583 | 0.99 (0.90-1.09) | 0.82 |  |  |  |
| Breast | MDS | per 1 tertile change | 407 | 1.08 (1.02-1.14) | 0.01 |  |  |  | 407 | 1.08 (1.02-1.14) | 0.01 |
| GI | MDS | per 1 tertile change | 378 | 1.05 (0.97-1.13) | 0.22 | 215 | 1.08 (0.92-1.26) | 0.33 | 163 | 1.04 (0.96-1.14) | 0.34 |
| CRC | MDS | per 1 tertile change | 234 | 1.04 (0.94-1.14) | 0.43 | 130 | 1.09 (0.89-1.33) | 0.42 | 104 | 1.03 (0.92-1.15) | 0.58 |

**S3 Table.** Hazard ratios (HRs) and 95% CI for longitudinal change in DII and MDS for all cancer and the most common types of cancers, demonstrating that the positive association between MDS and cancer was primarily driven by breast cancer in women for those who had changed their diet towards a better adherence to a Mediterranean diet.
