# Supplementary material for: The inflammatory potential of diet in determining cancer risk; A prospective investigation of two dietary pattern scores
Source: PLoS One. 2019 Apr 12;14(4):e0214551. doi: 10.1371/journal.pone.0214551 (PMC6461253; doi:10.1371/journal.pone.0214551)
Supplement: S1 Fig — Hazard ratios (HRs) and 95% CI for all cancer per tertile decrease in DII and tertile increase per MDS at baseline. (DOCX) [file pone.0214551.s004.docx]

**S1 Fig.** Hazard Ratios (HRs) and 95% CI for cancer per tertile decrease in DII, and tertile increase in MDS, at baseline (n=96,870), excluding participants with self-reported diabetes or diabetes diagnosed at the health examination (n=4011). HRs obtained from Cox regression using age as the time scale. Dietary pattern variables were included as continuous variables scaled by dividing by the sex and FFQ-specific intertertile ranges. Mean intertertile range were: DII = 1.7, MDS = 2. Estimates marked in gray had a potential non-linear association, illustrated in Fig 3. Estimates were adjusted for energy intake, BMI, physical activity, smoking, educational status, and, in the all participant analyses, stratified by sex in the Cox model. Predictive accuracy (C-index) for cancer for each model were calculated using predictions with the Cox regression models using ten-fold cross-validation. The C-index is measured on a scale from 0.5 to 1, where 0.5 corresponds to a prediction accuracy no better than guessing and 1 corresponds to a perfect prediction
