# Supplementary material for: The inflammatory potential of diet in determining cancer risk; A prospective investigation of two dietary pattern scores
Source: PLoS One. 2019 Apr 12;14(4):e0214551. doi: 10.1371/journal.pone.0214551 (PMC6461253; doi:10.1371/journal.pone.0214551)
Supplement: S2 Fig — (DOCX) [file pone.0214551.s005.docx]

**S2 Fig.** Hazard ratios (HRs) and 95% CI for cancer (all types) per tertile decrease in DII, and per tertile increase in MDS, at baseline in subgroups defined by age at study entry (VIP age groups ±2 years), smoking status, and BMI. HRs obtained from Cox regression using age as time scale. Dietary pattern variables were included as continuous variables scaled by dividing by their respective sex- and FFQ-specific intertertile ranges. Estimates were adjusted for energy intake, BMI, physical activity, smoking, and educational status. *P* for heterogeneity were obtained by Wald tests of equal HRs across subgroups.

Abbreviations: BMI, body mass index; CI, confidence interval: DII, Dietary inflammatory index; FFQ, food frequency questionnaire, MDS: Mediterranean dietary score; HR, Hazard ratios
