# Supplementary material for: The inflammatory potential of diet in determining cancer risk; A prospective investigation of two dietary pattern scores
Source: PLoS One. 2019 Apr 12;14(4):e0214551. doi: 10.1371/journal.pone.0214551 (PMC6461253; doi:10.1371/journal.pone.0214551)
Supplement: S3 Fig — (DOCX) [file pone.0214551.s006.docx]

**S3 Fig.** Hazard ratios (HRs) and 95% CI for smoking-related and obesity-related cancer per tertile decrease in DII, and tertile increase per MDS, at baseline in subgroups defined by smoking status and BMI. HRs obtained from Cox regression using age as time scale, and stratified on sex. Dietary pattern variables were included as continuous variables scaled by dividing by sex and FFQ specific intertertile range. Estimates were adjusted for energy intake, BMI, physical activity, smoking, and educational status. *P* for heterogeneity are from Wald’s tests of equal HRs across subgroups.

Abbreviations: BMI, body mass index; CI, confidence interval; DII, Dietary inflammatory index; FFQ, food frequency questionnaire; MDS, Mediterranean dietary score; HR, Hazard ratio.
