# Supplementary material for: The inflammatory potential of diet in determining cancer risk; A prospective investigation of two dietary pattern scores
Source: PLoS One. 2019 Apr 12;14(4):e0214551. doi: 10.1371/journal.pone.0214551 (PMC6461253; doi:10.1371/journal.pone.0214551)
Supplement: S4 Fig — Restricted cubic splines with hazard ratio (HR) and 95% confidence interval of cancer in a) all participants b) men, and c) women by baseline dietary pattern score. (DOCX) [file pone.0214551.s007.docx]

| **A**  ****  **B**  ****  **C**  **** |
| --- |
|  |

**S4 Fig.** Hazard ratio (HR) (black line) and 95% confidence interval (gray area) of cancer in **a)** all participants **b)** men, and **c)** women by baseline dietary pattern score. HRs were calculated with restricted cubic splines (with knots on the 5^th^, 50^th^, and 95^th^ percentiles) in Cox regression models using attained age as the time scale. Presence of an association was tested with a likelihood ratio test comparing the dietary pattern spline model to a model without dietary pattern. Nonlinearity was tested using a likelihood ratio test comparing the spline model to a linear model. The HRs were adjusted for energy intake, BMI, physical activity, smoking, and educational status.

Abbreviations: CI, confidence interval; DII, Dietary inflammatory index; FFQ, food frequency questionnaire; GI, Gastrointestinal; MDS, Mediterranean dietary score; HR, Hazard ratio.
