# Supplementary material for: The inflammatory potential of diet in determining cancer risk; A prospective investigation of two dietary pattern scores
Source: PLoS One. 2019 Apr 12;14(4):e0214551. doi: 10.1371/journal.pone.0214551 (PMC6461253; doi:10.1371/journal.pone.0214551)
Supplement: S5 Fig — (DOCX) [file pone.0214551.s008.docx]

**S5 Fig.** Distribution of 10-year longitudinal changes in dietary patterns. Categorical variables were defined according to baseline and repeat values on dichotomous dietary pattern variables (“unhealthy” defined as DII 3^rd^ tertile and MDS 1^st^ tertile, using sex and FFQ specific cut-offs).
